# An Efficient Closed-Loop Adaptive Controller for a Small-Sized Quadruped Robotic Rat

**DOI:** 10.34133/cbsystems.0096

**Published:** 2024-05-27

**Authors:** Xiaolong Quan, Rongjie Du, Ruochao Wang, Zhenshan Bing, Qing Shi

**Affiliations:** ^1^ Intelligent Robotics Institute, School of Mechatronical Engineering, Beijing Institute of Technology, Beijing 100081, China.; ^2^ Key Laboratory of Biomimetic Robots and Systems (Beijing Institute of Technology), Ministry of Education, Beijing 100081, China.; ^3^ Technical University of Munich, Munich, Germany.

## Abstract

Large quadruped robots have shown potential for a wide range of everyday tasks due to their superior terrain adaptation. However, small-scale quadruped robots have limited payloads and thus cannot carry sufficient sensing and computational resources, which imposes limitations on their environmental adaptability. To address this challenge, we proposed an efficient closed-loop adaptive controller by simplified pose estimation and control strategy that utilizes only inertial measurement unit sensors, which drastically reduces the control computation. Accordingly, we integrated this control system into a small-scale quadruped robot, SQuRo, and conducted a series of experiments to verify the environmental adaptation performance of SQuRo. The results demonstrated that SQuRo has achieved 6 kinds of robust motion: slope stabilization motion, linear tracking motion, autonomous fall recovery motion, uneven terrain walk, slope walk, and obstacle avoidance. This work paves the way for small-scale quadruped robots to autonomously perform tasks in challenging environments.

## Introduction

By autonomously selecting contact points with the environment, legged robots can overcome obstacles equivalent to leg length, so in the application of rough terrain and complex and chaotic environment, legged robots are effective substitutes for tracked/wheeled robots and have broad application prospects. Legged robotic systems have the potential to perform any physical activity that humans and animals are capable of and in the future may be used in rescue operations in forests and mountains, carrying payloads for transport in scenarios such as construction sites, inspecting unstructured underground tunnels, and exploring other planets. As a hot spot of legged robot research, quadruped robot currently has a wide range of application scenarios, which can undertake the transportation of materials in dangerous areas in military missions, as well as signal monitoring, regional reconnaissance, factory inspection and other reconnaissance tasks. However, the application of quadruped robot in narrow space is very hindered.

Narrow space exploration such as pipeline detection, debris survey, disaster relief, and so on are extremely difficult and dangerous tasks, which contain many inevitably challenging environments. At present, there are few robots that can reliably complete the task of narrow space exploration because of unknown operating danger, small working space, and complex environment in narrow space. Large 4-legged robots such as MIT Cheetah and ANYmal have strong transportation capabilities, but their size is too large to work in narrow spaces [1–3]. Some small-scale quadruped robots weighing in the Arc class have small size and can enter narrow spaces, but their carrying capacity is very weak and it is difficult to effectively operate in narrow spaces [4–6]. Therefore, the development of a centimeter-long small-scale quadruped robot with a load capacity of several hundred grams can effectively solve the above problems. However, the existing small-scale quadruped robots cannot meet the needs of multiterrain adaptation, which seriously affects the environmental adaptability of robots and leads to their inability to complete narrow space tasks.

As one of the small-scale quadruped robots, SQuRo also faces the problem of difficult control of small-scale robots. As a highly dynamic system, small-scale quadruped robots are hard to be equipped with a variety of sensors such as force sensor, radar, and high-definition camera [7,8] like large quadruped robots due to scale and weight limitations, and they cannot be equipped with high-performance processors. A large quadruped robot can establish a 3-dimensional elevation map with rich environmental information through rich external sensing modules [3,9–12] and simultaneously calculate the complex dynamics of the robot body and the optimization problem of the controller through high-speed computing units. For example, MIT Cheetah 2 can control the robot to cross obstacles through a fast online controller [13]. MIT Cheetah 3 through online convex model predictive control for dynamic walking [14]. Joonho Lee et al. [3] modeled the controller as a neural network policy trained by reinforcement learning in their simulation, which achieved walking on a variety of terraforms. These complex dynamics solving and optimization problem solving will consume a lot of computing resources, and some early quadruped platforms even installed 6 control boards to realize real-time control [15]. However, due to the lack of multiple sensors and high-performance central processing units, the existing small-scale quadruped robots have not yet shown the robustness and environmental adaptability commensurate with the same type of large quadruped robots, which also greatly hinders the application of small-scale quadruped robots in scenarios such as narrow spaces. The difficulty lies not only in the mechatronics design but also in the design of the control strategy. Under the size limitation, small-scale quadruped robots are rarely equipped with inertial measurement units (IMUs) and cameras, and the embedded central processing unit suitable for small-scale quadruped robots is difficult to process data and run the controller at the same time. Therefore, efficient closed-loop motion control under limited resources is difficult.

To conquer this problem, a lot of work has been completed on installation of sensors in small-scale quadruped robot [16–17]. This paper is extended from the conference version [18] by adding an efficient closed-loop adaptive motion controller for SQuRo to autonomously perform tasks in challenging environments. In our conference paper, we proposed a posture estimation and trajectory tracking method which enable SQuRo to perform well in certain kinds of state. We found that by integrated IMU, SQuRo can autonomously perform more tasks in challenging environments.

Based on the multimodal motion controller, an adaptive motion controller for different situations is established by using the feedback attitude Angle information from IMU module in real time. The controller also follows the goal of designing the controller to be as efficient as possible with the goal of improving environmental adaptability. The state detection module and adaptive motion generation module are designed on the basis of multimodal motion controller. This method based on state detection and corresponding adaptive motion generation avoids solving the global optimization problem. Thanks to the optimized basic motion modes, the adaptive motion module in this paper will mainly use the transformation of basic motion modes and predefined operating sequences to cope with different environments, so there is no need to generate real-time motion but to select from a series of predefined actions, similar to the idea of a state machine [19–20].

In the state detection, we selected fall, complex terrain, slope, and collision. For each case, the feedback data of the IMU module will show different characteristics. Therefore, we can identify specific scenarios through the characteristics of IMU data. In the adaptive motion generation, the corresponding adaptive motion is designed to overcome the current situation according to different situations and features that can be recognized by IMU.

In Efficient closed-loop adaptive motion control, we propose an adaptive motion controller which makes full use of the optimized basic motion modes. In Posture estimation algorithm, we establish the kinematic model, solve the relationship between the foot position and the servo motor, and introduce the pose calculation strategy. In Motion control strategy, we expound the fall in the attitude control strategy, trajectory tracking algorithm, independent testing and detection and recovery, uneven terrain modal transformation, slope test and modal transformation as well as collision detection and obstacle avoidance. Six different experiments are conducted in Experiments and analyses to validate the proposed method and evaluate the robustness enhancement of the SQuRo.

## Materials and Methods

### Efficient closed-loop adaptive motion control

#### Controller design

**Fig. 1.**
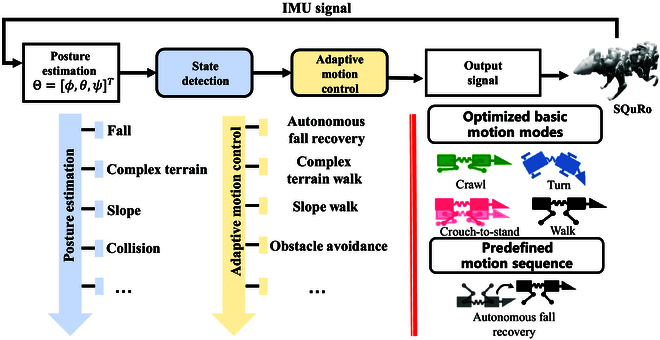
The schematic illustrates the design of the controller.

The design idea of the controller can be summarized as follows: by analyzing the IMU signal, state detection is performed to judge the situations faced by SQuRo, and a response strategy is specially designed for each situation. In order to achieve efficient control, these strategies are preprogrammed and optimized transitions between basic motion modes in the main control unit and artificial action sequences designed to leverage SQuRo’s design advantages. Figure 1 is the schematic diagram of controller design. When the state detection module recognizes a fall, the adaptive motion generation module will perform an autonomous fall climb. Similarly, slope walking (mode switching) is performed when slopes are encountered, and complex terrain walking (mode switching) is performed when complex terrain is encountered. The high efficiency of the controller is reflected in the following aspects:

1. The basic motion mode of offline optimization is directly adopted during movement, which ensures the effect of movement and avoids the optimization calculation on the line.

2. IMU module built-in Kalman filter program, the main control unit can directly process the filtered attitude angle, save a certain signal preprocessing process.

3. The adaptive motion is closely combined with the basic motion mode of offline optimization, and the motion mode can be easily changed to cope with a variety of scenarios.

4. The controller can design efficient and reliable action sequences for other situations that require adaptive motion of complex action sequences to avoid real-time calculation optimization problems.

After the state detection module and adaptive motion generation module are included in the multimodal motion hierarchical control block diagram, the complete motion controller of SQuRo motion will be obtained, as shown in Fig. 1. The offline optimization part mainly completes the optimization of basic motion modes, enabling SQuRo to get rid of the large computational burden brought by traditional optimization-based controllers, and the data-driven method also captures SQuRo’s dynamic characteristics well. The online program includes gait pattern adjuster, joint trajectory generator, attitude estimation, state detection, and adaptive motion control. Among them, the gait pattern regulator and joint trajectory generator are mainly responsible for mapping the change of control variable χ to joint trajectory, which is the lowest level of SQuRo motion control. The state detection and adaptive motion generation modules identify the situation through IMU signal analysis and carry out targeted adaptive movements, and these adaptive movements will also be carried out through the change of control variables. Other predefined preprogrammed adaptive movements will be described in detail later in the article.

#### Hardware system

**Fig. 2.**
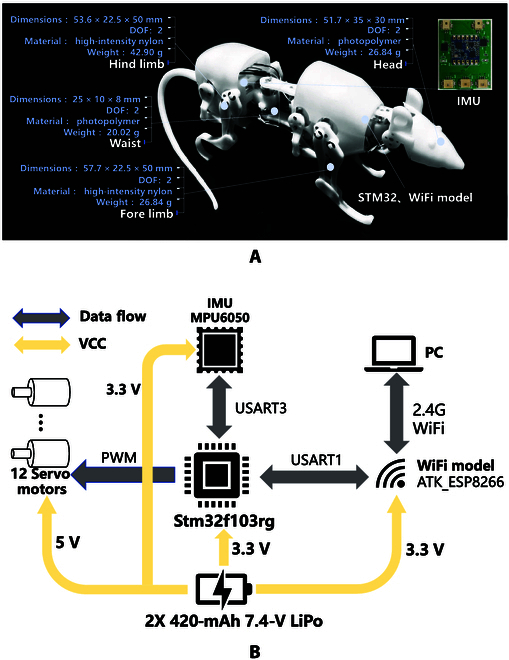
Overview of SQuRo system. (A) Hardware system. (B) circuit system.

Two compact control panels are used to drive 12 servo motors. Two control panels are mounted at the front (main) and rear (slave) of the robot’s spine, as shown in Fig. 2A. In this paper, the IMU has been installed in the circuit system, so that the robotic rat can receive information feedback and determine the robotic rat’s own posture, so as to realize the posture estimation and trajectory tracking. For the circuit connection presented in Fig. 2B, the STM32F103RG microcontroller is connected to the IMU module MPU6050 through the USART serial port. At the same time, the WiFi module is also connected to the microcontroller through another USART, which can be used to remotely control the robotic rat, and at the same time, it is used to return the data such as the status of the robotic rat for easy debugging. The operation time of the robot is about 25 min, which is an important feature for effective detection in challenging environments.

### Posture estimation algorithm

State detection is an important part of adaptive motion control, which is responsible for observing the state of the robot during the process of SQuRo and identifying the environment of the robotic rat through feature matching to prepare for the generation of adaptive motion in the future.

First, we set an inertial coordinate system in our laboratory, then fix a body coordinate on robotic rat, whose zero point is the center of mass (CoM) of the robot, moving along with robotic rat.

According to the general transform matrix

x3=x0+l1cosθ1+l2cosθ1+θ2y3=y0+l1sinsθ1+l2sinθ1+θ2 (1)

We can first calculate the 2 solutions of *θ*_2_, and we can know that the positive and negative 2 solutions of *θ*_2_

$$\theta_{2}=\pm \;\text{arccos}\left(\frac{l_{2}^{2}-l_{1}^{2}-x^{2}-y^{2}}{2l_{1}l_{2}}\right)$$ (2)

Since the previous limbs are examples, the *θ* negative, rewrite the equal form of *x*, *y*.

$$\left\{\begin{array}{c} x=l_{1}c_{1}+l_{2}\left(c_{1}c_{2}-s_{1}s_{2}\right)\\ y=l_{1}s_{1}+l_{2}\left(c_{1}s_{2}+s_{1}c_{2}\right) \end{array}\right.\;$$ (3)

Change the parameters: *x* = *k*_1_*c*_1_ − *k*_2_*s*_1_, *y* = *k*_1_*s*_1_ + *k*_2_*c*_1_, *k*_1_ = *l*_1_ + *l*_2_*c*_2_, *k*_2_ = *l*_2_*s*_2_

$$\text{Let}\;r=\sqrt{k_{1}^{2}+k_{2}^{2}},\gamma =\;\text{arctan}\left(\frac{k_{2}}{k_{1}}\right),k_{1}=\;r\text{cos}\gamma ,k_{2}=\;r\text{sin}\gamma$$

$$\theta_{1}=\text{arctan}\left(\frac{y}{x}\right)-\text{arctan}\left(\frac{k_{2}}{k_{1}}\right)$$ (4)

The transformation matrix of the foot to the body coordinates is

$${\;}_{0}^{-1}R=R_{x}\left(90^{\circ }\right)R_{y}\left(0^{\circ }\right)R_{z}\left(-\;90^{\circ }\right)=\left[\begin{array}{ccc} 0 & -1 & 0\\ 0 & 0 & 1\\ -1 & 0 & 0 \end{array}\right]$$ (5)

The homogeneous transform matrix is

$${\;}_{3}^{-1}T={\;}_{0}^{-1}T_{3}^{0}T=\left[\begin{array}{cccc} -s_{12} & -c_{12} & 0 & \frac{1}{2}\mathrm{BL}-s_{1}a_{1}-s_{12}a_{2}\\ 0 & 0 & -1 & \frac{1}{2}\mathrm{BW}\\ -c_{12} & s_{12} & 0 & -c_{1}a_{1}-c_{12}a_{2}\\ 0 & 0 & 0 & 1 \end{array}\right]$$ (6)

According to the half body length of the robotic rat, the height difference between the shoulder joint and the center of mass (*CoM*), ${\;}_{\;3}^{-1}T$ can be determined, so that the position of the left foot end is known. Determined through the input angles of the servo motor, in the same way, we can calculate the spatial description of the other 3 foot ends under the body coordinates [21]. [*p*1, *p*2, *p*3, *p*4 ]*^T^* is the 3-dimensional column vector under the body coordinate system, so we can get:

pFL=12BL−s1a1−s12a212BW−c1a1−c12a2pFR=12BL−s1a1−s12a2−12BW−c1a1−c12a2pHF=−12BL−s1a1−s12a212BW−c1a1−c12a2pHR=−12BL−s1a1−s12a2−12BW−c1a1−c12a2 (7)

### Motion control strategy

The adaptive motion module receives the message from the status detection module and is responsible for executing the corresponding adaptive motion. This chapter mainly analyzes the changes of robot rat posture in various scenarios and how to extract basic features in different scenarios through IMU signals and introduces the corresponding program implementation. Each corresponding adaptive motion is also described in detail in this section.

#### Posture control strategy

The advantage of legged robots is their walking ability under uneven terrain [22]. In natural environment, slope is a very common terrain [23]. The robot must have a certain off-road capability to achieve stability on the slope surface. Regarding the stability of the robotic rat posture, there are 2 main tasks facing the robotic rat: one is how to perceive the terrain and provide terrain information for the robot movement; the other is how to plan the foot-end location online according to the terrain. In this work, we will use the robot joint angles to fit on the slope. In order to accurately respond to the slope angle, the Kalman filter was used to filter the posture angle data, and the joint angles of the robot’s legs were planned online to keep the robotic rat stable on the slope [24]. The angle of slope surface can be estimated by the IMU data of the body and foot ends on the support surface; meanwhile, the body posture rotation matrix is estimated by computer in the slope estimation.

$${\;}_{b}^{r}R=\left[\begin{array}{ccc} \text{cos}\alpha & \text{sin}\alpha \text{sin}\phi & \text{cos}\phi \text{sin}\theta \\ 0 & \text{cos}\phi & -\text{sin}\phi \\ -\text{sin}\alpha & \text{cos}\alpha \text{sin}\phi & \text{cos}\alpha \text{cos}\phi \end{array}\right]$$ (11)

**Fig. 3.**
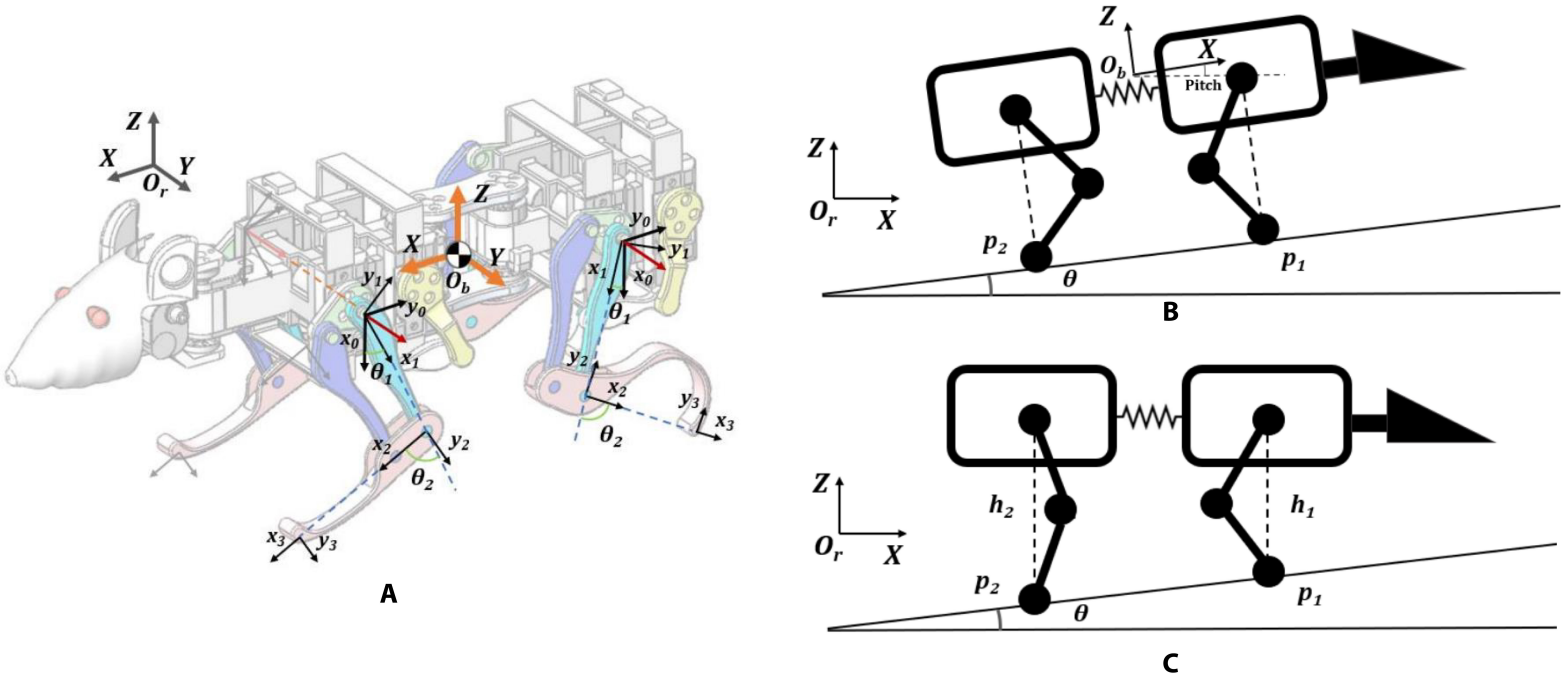
The kinematic model of SQuRo. (A) The coordinate system of SQuRo. (B) The robot on the slope before posture adjustment. (C) The robot on the slope after posture adjustment.

In this way, the body frame {𝑂} relative to the posture of the world frame {𝑂}, which is the gesture of the robot body. Combined with the posture of the robotic rat and the kinematic information of the foot, the slope of the unknown slope can be calculated, and the posture of the robot can be adjusted to achieve balance control of stance on the slope. When the robotic rat perceives the slope changes of the terrain, they can combine the robot legs kinematic model to fit the slope terrain. When the slope of the robot support surface changes, the body’s posture will inevitably change with the support surface. The schematic diagram of the robot on the slope is shown in Fig. 3B.

𝑝1 and 𝑝2 are the positions of the feet. In the figure, only the position of the leg 1 or 2 is marked, leg 3 or 4 are not. The coordinates of the position of the supporting leg in the body coordinate system are pib=xib,yib,zibt , here 𝑖 = 1,2,3,4. Foot coordinates in the world frame {*O*}*r* can be obtained from the following formula

$$p_{i}^{r}={\;}_{b}^{r}R\cdot p_{i}^{b}$$ (12)

Here, pir=xir,yir,zirT . According to the principle of geometry, in the world coordinate system, any 3 points can determine a plane, this plane equation is:

Ax+By+Cz=1 (13)

Here, 𝐶 ≠ 0, otherwise if 𝐶 = 0, the equation is *Ax* + *By* = 1, at this time, the plane is parallel to the Z axis. That is, a wall that is perpendicular to the ground, this is impossible, unless the robotic rat is rolling over. Take any 3 foot-end positions in kinematic model, the coordinates of them are p1r=x1r,y1r,z1rT, p2r=x2r,y2r,z2rT, p3r=x3r,y3r,z3rT, since the foot-end positions are located on the support plane, satisfying Eq. 13, thus we can get

xr1yr1zr1xr2yr2zr2xr3yr3zr3ABC=111 (14)

These vectors will not lie on the same plane because of the robotic rat mechanism; thus, the parameter matrix is inversable, the parameters of supporting plane are

ABC=xr1yr1zr1xr2yr2zr2xr3yr3zr3−1111 (15)

The Eq. 13 can be rewritten:

$$z=1-\frac{A}{C}x-\frac{B}{C}y$$ (16)

$$\nabla z={\left[\begin{array}{cc} \frac{\partial z}{\partial x} & \frac{\partial z}{\partial y} \end{array}\right]}^{T}={\left[\begin{array}{cc} -\frac{A}{C} & -\frac{B}{C} \end{array}\right]}^{T}$$ (17)

The modulus of the gradient is the size of the slope, that is,

$$∥\nabla z∥=\frac{\sqrt{A^{2}+B^{2}}}{C}$$ (18)

$$\begin{array}{c} \beta \;=\;\text{arctan}∥\;\nabla z\;∥\;=\;\text{arctan}\frac{\sqrt{A^{2}+B^{2}}}{C} \end{array}$$ (19)

When the quadruped robot standing on the slope, due to the changes in the support surface, the force of the robot will also change. When the robot is uphill, the distance between the projection point of the backward support point and the center of gravity in the vertical direction is closer. Therefore, the force of the hind legs is greater than the force of the front leg, and most of the robot’s weight is supported by the hind legs; when the robot is downhill, the distance between the front leg support and the focus of gravity in the vertical direction is closer, so the force of the front leg is greater than the force of the hind legs. Most of the robots are supported by the front leg. In addition, when the robot is walking on the slope, the stability of the robot becomes lower, that is, the shortest distance of the projection point of the center of gravity is reduced. To make the robot’s front and hind legs be balanced and increase the stability of the robot, the robot needs to adjust the body’s posture based on the slope to keep the robot stable on the slope [25]. It can be seen through observation of rats in nature that when rats are moving uphill or downhill, they will lean forward and backward in the body accordingly. The body posture adjustment method is shown in Fig. 3C.

The forelimbs and hind limbs are exactly the 2 different solutions obtained by the double-link robotic movement [26]. Since the joint angle of the machine rat on the slope and the left and right limbs is the same, the calculation principle of the front and back limbs is the same, however the symbols of angles are different (*θ*_1_ > 0, *θ*_1_ < 0)

pFR=12BL−s1l1−s12l2−12BW−c1l1−c12l2=12BL−12BW−h1 (20)

$$∆h(\psi )=h_{2}-h_{1}=BL\cdot \tan(\;\psi \;)$$ (21)

θ2=arccosl22−l12−h122l1l2θ1=−arctanl1+l2c2l2s2 (22)

θ2=−arccosl22−l12−h122l1l2θ1=arctanl1+l2c2l2s2 (23)

#### Trajectory tracking algorithm

In order to precisely track the trajectory through which it passes, a common method is to research and analyze its path [27]; sometimes, some practical requirements are considered, such as the maximum output torque limit of the motor, the minimum time to complete the task, the minimum energy consumption to complete the task, etc.

The analysis and research of the movement trajectory of the mobile robot is based on the positioning of the mobile robot. If the position cannot be located precisely, the trajectory will lose its practical significance; in the process of using the navigation of the mobile robot for the planned trajectory, if the mobile robot control cannot be effectively implemented, it will lose its application value as well. The representation of the environment model and the positioning based on IMU allow the robot to complete most of the trajectory.

In an unknown environment, the surrounding environmental information is very vague for the robotic rat. If only relying on the internal sensors of the mobile robot to obtain the robot’s own state information, it is easy to accumulate errors due to the lack of environmental information [28].

It can be said that once the path of the mobile robot’s movement is determined on the basis of its movement route in the entire environment map [29]; after that, the trajectory of the robotic rat can be solved via kinematic model and dynamic analysis, and then all its variables containing time attributes (such as displacement, velocity, acceleration, motor output torque etc.) will be determined, that is, we obtain the form of motion along the path which is our desired trajectory. It is easy to find that path planning is the basis of trajectory planning, and trajectory planning is a further study of path planning. No matter what kind of navigation method is adopted, the robotic rat mainly completes basic tasks such as path planning, positioning, and obstacle avoidance when performing tasks. Path planning means that a mobile robot plans and searches for an optimal or suboptimal collision-free path from the initial state to the target state according to a certain performance index (such as the shortest path, the least time-consuming, the lowest energy consumption, etc.). Trajectory planning is based on path planning and generates motion time series under the premise of satisfying system constraints.

In mobile robot path planning, it is generally not necessary to consider the dynamic characteristics of the mobile robot [30]. At this time, only its geometric characteristics need to be considered, and its dynamic characteristics need not be considered, such as the shortest path, the best obstacle avoidance, the least in the case of time-consuming, etc., for the planning of the lowest energy consumption; when the path is the shortest and the time consuming is the least, the energy consumption is generally the least. To simplify the analysis, the robot can be regarded as a particle with a speed vector.

#### Fall detection and autonomous recovery

Under normal circumstances, the controller of quadruped robot takes the stability of moving as one of the control objectives, but quadruped robot is often prone to capsize and fall when it passes through complex roads and encounters external shocks [31]. However, small-scale quadruped robots basically do not have the ability to stand up from the fall in, which limits their practical application. In order to achieve postfall recovery, the first thing that needs to be done is fall detection.

**Fig. 4.**
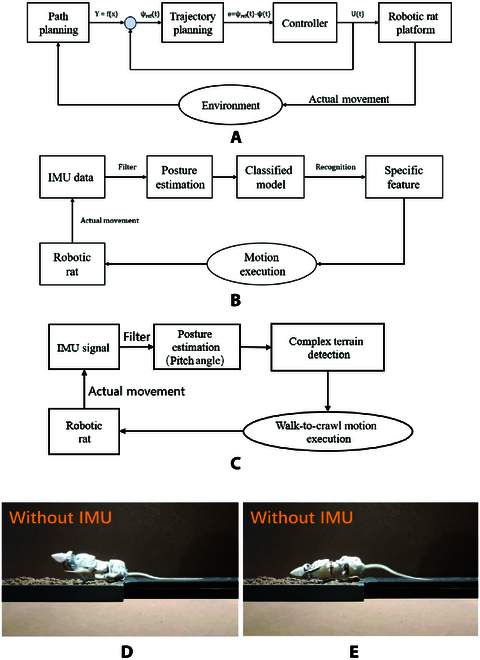
Control diagrams of SQuRo. (A) Trajectory tracking. (B) Autonomous recovery. (C) Complex terrain detection and adaptive motion modes switching. (D) and (E) depict snapshots of walking on uneven terrain without theuse of IMU.

It is easy to understand that the roll angle of SQuRo will change greatly when falling, and the IMU signal shows a rapid and large roll angle change. Therefore, we complete the fall detection by detecting the sharp change of the roll angle in the IMU signal. Specifically, when the roll angle of SQuRo is greater than 60° for all 4 data return cycles (20 ms), SQuRo is determined to be in a falling state. Among them, a 60° increase in roll angle results in a right-handed tilt, while a 60° decrease in roll angle results in a left-handed tilt. The control block diagram can be expressed as Fig. 4B. After detecting the fall, SQuRo can then execute the fall recovery motion model we proposed in our previous study to achieve autonomous fall detection and recovery during locomotion [26].

#### Complex terrain detection and adaptive motion modes switching

Legged robots often encounter unstructured terrain, such as grass, snow, sand, stone, and muddy ground, when performing tasks. How to ensure stable walking in such an environment is also a hot issue in the field of quadruped [3,32]. Aiming at this problem, this study makes full use of the structure characteristics of SQuRo and optimization of multimodal movement to complete the stability of the complex terrain of walking. Without adaptive control of complex terrain, SQuRo will struggle to walk on uneven ground, as shown in Fig. 4. When SQuRo walks on a muddy surface, the foot end is easy to fall into it (Fig. 4A) and cause rollover (Fig. 4B).

In order to overcome the above situation, the need for complex terrain is identified in the first place. In general, when the same mode is used to walk on complex terrain, the pitch angle and pitch rate will change drastically, and the stability of the fuselage will become worse. In addition, the phase diagram composed of pitch angle and pitch angle rate is often used to characterize the dynamic system characteristics of quadruped robots. A smaller phase diagram of pitch angle and pitch angle rate generally indicates better stability [33]. Therefore, this study captured quadruped walking robot in complicated ground pitching angle change the fact that more intense, by detecting the pitch during a certain period of time of variance for state detection of complex terrain. Specific, when a gait cycle of pitching angle variance greater than 5°, think bot is walking on the complicated terrain. In order to be able to walk in complex terrain, this study makes full use of the high stability of the prostrate mode. When dealing with complex terrain, the movement mode conversion from straight to crawling is adopted, that is, the body height z is lowered, and the CoM is reduced. In addition, the foot end creeping condition on the ground contact area, also can effectively avoid the foot end into the ground. At this time, the control block diagram is shown in Fig. 4C.

#### Slope detection and adaptive slope walk

**Fig. 5.**
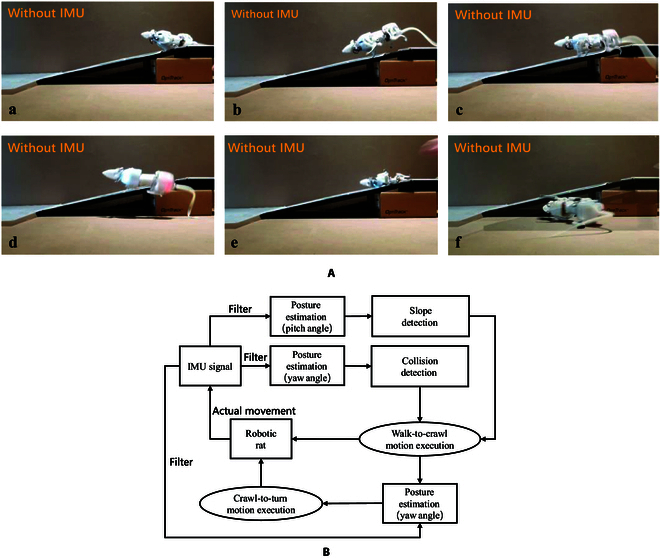
(A) Experiments of slope walking. Snapshots depict SQuRo walking on a slope without the use of IMU. (B) Control diagram of slope detection and adaptive slope walk; collision detection and obstacle avoidance.

In addition to complex terrain, it is common to encounter rough terrain such as slopes, but there are dramatic changes in the ground. When the slope is not effectively detected, the robot will often fall or produce a wrong difference in the direction angle. Take the controller without feedback used by SQuRo as an example, as shown in Fig. 5A. In the transition from the ground to the slope, due to the sudden loss of height, a variety of unexpected conditions can occur, such as: In one gait cycle, the support phase did not effectively contact the slope (Fig. 5Aa), the fuselage produced a large capsize, resulting in severe fluctuations of the hind limbs (Fig. 5Ab), and the hind limbs stepped on the ground at the slope interface (Fig. 5Ac) and fell (Fig. 5Ad and e), and unnecessary falling (Fig. 5Af) due to the lack of control of the forward direction.

For this reason, this study firstly analyses the IMU signals passing through the slope and finds that there will be a single sharp and rapid fluctuation in the pitch angle direction when passing through the slope, and there is also a high probability of generating the error in the direction angle (yaw angle) after passing through the slope due to the great interference received at the landing point. In the state detection, this study detects a single sharp and rapid fluctuation in the pitch angle as a judgement of whether to pass the slope or not. Specifically, the state detection procedure will detect whether there is a sudden change in the pitch angle of more than 15° in 4 data return cycles (20 ms), and if there is a sudden change, it is judged to have passed the slope; in addition, yaw angle error will be detected when passing the slope. It is worth noting that when the SQuRo is traveling on flat ground, the IMU signals indicate that the yaw error will be cancelled by using a PD controller pair, whose outputs will correspond to changing the left and right side step sizes.

In account of the slope, the adaptive motion like walking complex terrain, is first to ensure stability, so the first adaptive motion is walk-to-crawl mode switch motion. This is because on slopes, SQuRo has a lower center of gravity and greater stability during crawl motion. In addition, when the slope encountered yaw angle error, the form of creeping turn will be adopted, that is, the waist angle φ will be adjusted under the condition of reducing the body height z, to realize a stable and positive small radius turn. At this point, the block diagram is shown in Fig. 5B. In the process of slope recognition and walking, the pitch angle was used to detect whether the robotic rat encountered a slope. If it encountered a slope, the motion mode was changed into a more stable prostrate mode. In addition, when yaw angle error is also detected on the slope, the turning mode is used to adjust the direction. It is worth noting that when the robotic rat walks on the slope, its pitch angle will keep fluctuating around the slope angle, and when the robotic rat walks the slope, the pitch angle will return to the fluctuation around 0°. According to the change of the pitch angle, it can also be judged whether the slope is finished. After walking the ramp, the robotic rat can return to its normal straight motion.

#### Collision detection and obstacle avoidance

In addition, robots often encounter external shocks while performing tasks. Without effective impact detection and avoidance, the robot will often be blocked or further damaged.

Therefore, this study first describes SQuRo external shocks of IMU signal analysis and found aftershocks with last severe rapid fluctuations in the yaw angle direction. In terms of state detection, this study uses a single sharp fluctuation in yaw angle as the basis for determining whether the robot has been impacted or not. Specifically, the state detection procedure detects whether the change in yaw angle is greater than 20° within 4 data return cycles (20 ms), and if it is greater than 20°, then SQuRo is determined to be in the state of being impacted. In addition, the specific change direction of the yaw angle will be detected when passing through the impact. To put it simply, after the robot detects the mutation of its yaw angle through IMU, it calculates the collision direction by the size and direction of the yaw angle before and after the mutation and gives the opposite and collision avoidance direction, and the robot avoids the obstacles that have been collided by adjusting the running direction.

In the case of impact, obstacle avoidance is similar to walking on complex terrain, which is to ensure stability first. Therefore, the adaptive movement at this time is also a mode switch between straight and crawl. In addition, when the impact direction is obtained, the form of creeping turn will be adopted, that is, the waist angle φ will be adjusted under the condition of reducing the body height z, so as to realize a stable and positive small radius turn. At this point, the block diagram is shown in Fig. 5B.

In the collision detection and obstacle avoidance process, the yaw angle was used to detect whether SQuRo was in collision, and if it was in collision, the motion mode was changed into a more stable crawling mode. In addition, when the impact direction is obtained, the turning mode is used to adjust the direction to avoid the obstacle, so as to avoid further impact.

## Results

### Posture adjustment on a slope

Posture adjustment experiment is mainly to test the attitude adaptability adjustment ability of the robot to the real-time change of the environment levelness on the slope with adjustable slope. The robotic rat receives the data processed by IMU (MPU6050) and transmits posture data via WiFi to a PC or app, in order to debug and observe the performance. PC is responsible for monitoring result and display feedback value, sending control commands to the robotic rat driving system, then keep looping this process.

**Fig. 6.**
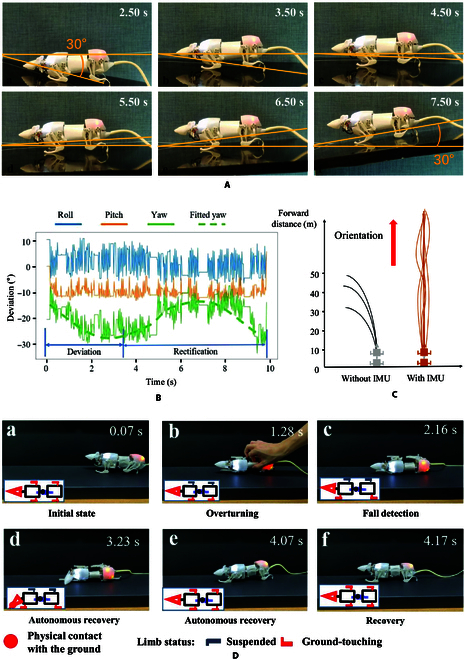
Experiments on posture adjustment, trajectory tracking, and autonomous recovery. (A) Snapshots depicting posture adjustment on a slope. (B) The posture of SQuRo during trajectory tracking. (C) A diagram illustrating 10 trials, where 3 were conducted without IMU, and 7 involved the use of the trajectory tracking algorithm. (D) Snapshots capturing the process of autonomous recovery.

In this experiment, we continuously manually adjusted the levelness of the slope on which the robot sited and observed the posture of the robot on the slope where the slope changed in real time. When the degree of slope increasing, the robotic rat adjusted joint angles to adapt to slope, so that it can keep balance without slipping. From the Fig. 6A, we can find that our robotic rat is able to sense the slope (step) and adjust the joint angles, which shows the strong adaptability in uneven terrain.

### Straight line tracking

After completing the hardware system of robotic rat, we checked the performance of every module, including IMU, WiFi connection, and STM32 on robotic rat, as well as mechanism, and then integrated them together. We can see that the trajectory tracking is successful.

As shown in Fig. 6B, the robotic rat transmits data during locomotion, and the average roll angle is not zero. Some subtle and unavoidable machining and assembly errors lead to bias when SQuRo walks in a straight line for a long distance in case without IMU. What needs to be noticed is that with the help of IMU, the robotic rat is able to rectify the yaw angle deviation, shown in Fig. 6B, so that it could track the initial orientation. The trajectory tracking effect of experiments is shown in Fig. 6C, and the deviation always happens without IMU; due to the trajectory tracking controller, the robotic rat has the ability to move in fix direction with small amount of vibration.

### Autonomous recovery

Due to the mechanical structure design of SQuRo, the most challenging fall accident that SQuRO may face during operation is complete rollover, which is also the most difficult state for the robot to recover to normal operation. During the experiment, in order to test the ability of SQuRo to fall and climb up autonomously, this study artificially overturned SQuRo at will and observed whether SQuRo could successfully fall and climb up autonomously. The experimental results are shown in Fig. 6D. State a is the normal walking state, and the limbs are in contact with the ground. State b for artificial capsized, near 2 side body contact with the ground, the far side limbs dangling. In state c, it can be seen that SQuRo actively controls the limb joint degree of freedom to extend forward of the front limb near the ground side and extend backward of the back limb, which means that it has detected that it has fallen into the fall-climb adaptive motion control. In state d, SQuRo controls the neck with 2 degrees of freedom so that the head is close to the ground. In state d to state e transformation, SQuRo control pitch degrees of freedom of the neck to the ground pressure and physical jacking, and fall in the control center of mass reaches the ideal position complete recovery. After that, the posture is adjusted to return to the normal state f where the limbs are in contact with the ground. By autonomously falling and climbing, SQuRo can improve its multiterrain adaptability and recover from falling state independently without human assistance. Other existing small-scale quadruped robots do not have the ability to autonomously fall and climb. SQuRo’s strategy of making full use of head DOF to complete falling and climbing can provide ideas for other robots with the same DOF configuration [26].

### Uneven terrain walk

**Fig. 7.**
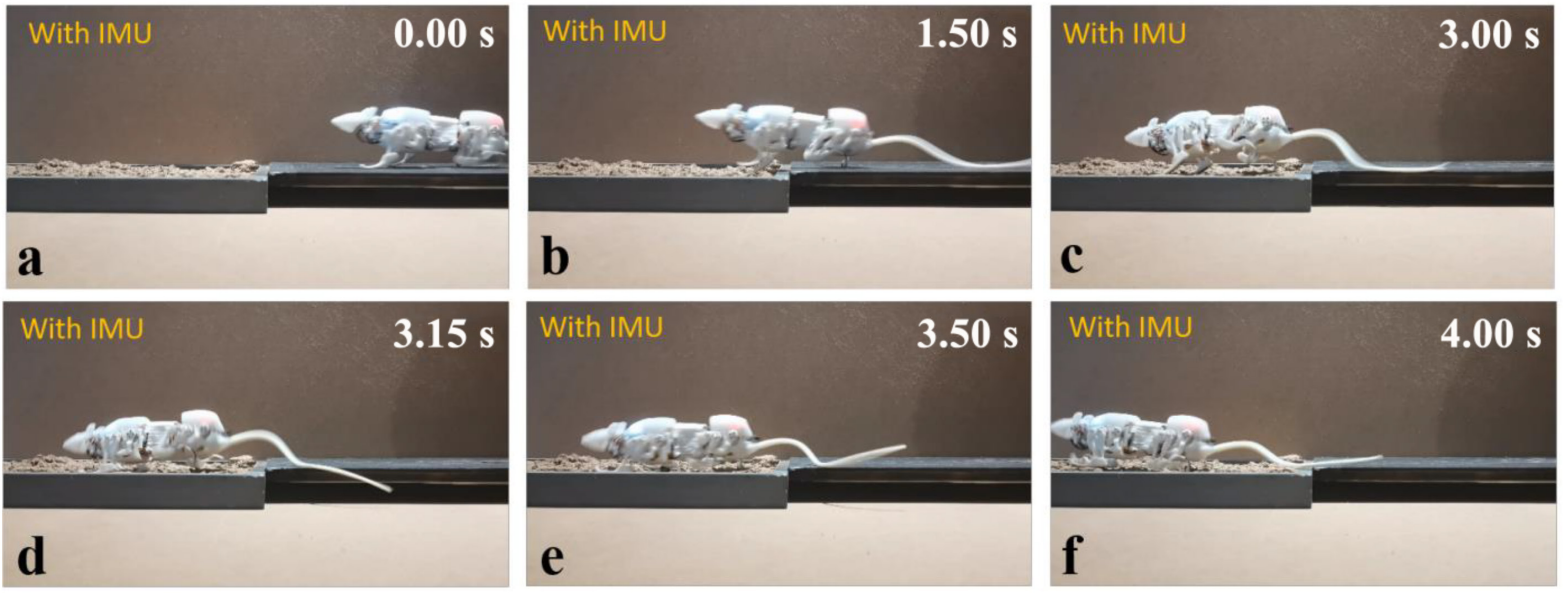
Experiments of walking on uneven terrain. Showcases snapshots of walking on uneven terrain with the assistance of IMU.

**Fig. 8.**
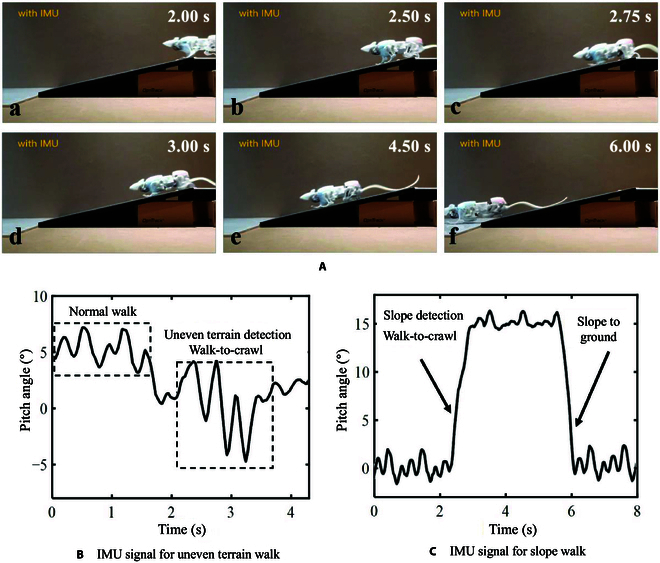
Experiments of slope walking and the corresponding IMU signals during the experiments. (A) Snapshots illustrate SQuRo walking on a slope with IMU assistance. (B) IMU signal recorded during uneven walking. (C) IMU signal recorded during slope walking.

The ability to adapt to uneven terrain walking is also crucial for movement in confined spaces. In order to test SQuRo’s feature recognition on uneven terrain and the completion of adaptive motion, this study took a self-made muddy road section as an example to test SQuRo’s trafficability. The test results are shown in Fig. 7. SQuRo entered the muddy road at around 1.5 s, ran straight on the muddy road from 1.5 to 3.15 s, completed the mode switch from straight to crawl from 3.15 to 3.50 s, and then passed the muddy road by crawling. The IMU data is shown in Fig. 8B. It can be seen from the figure that the pitch angle fluctuates little during normal walking (0 to 1.5 s) but fluctuates greatly under the muddy road section (2.0 to 3.5 s). At this time, the feature recognition module captures a large variance and implements adaptive motion: line-creeping mode switch. After mode switching, it can be seen that the IMU signal tends to be stable and the variance is markedly reduced. Through this experiment, SQuRo’s adaptability to the uneven terrain environment is verified by using an efficient closed-loop adaptive motion controller.

### Slope walk

Slopes also often occur in confined spaces. To test SQuRo’s ability to pass through a slope, a 15° ramp was constructed with wooden planks for testing. Since it has been verified in a previous paper that SQuRo possesses the ability to climb a 15° slope [26], this experiment focuses on the environmental adaptability of SQuRo when facing a road surface from flat to sloping, which is specifically denoted as the stability of walking on a slope. The experimental test process is shown in Fig. 8A. SQuRo is a straight movement of flat ground within 0 to 2.5 s. SQuRo made contact with the slope around 2.5 s, and it can be seen from IMU signal Fig. 8C that the pitch angle began to change at this time. Between 2.5 and 2.75 s, SQuRo recognized the slope characteristics through the drastic and large pitch angle change of IMU signal and completed the straight-crawling mode switch around 3.00 s. At 3.00 to 6.00 s, SQuRo crawled up the slope and walked down the slope at around 6.00 s, where the IMU signal showed that the pitch angle dropped from around 15° to around 0° at this time. As the main focus of this experiment is the robot’s forward direction and stability on the slope, the quantities considered in the implementation principle are the robot’s pitch and yaw angles, and these factors in the uphill and downhill experiments in the principle of the difference do not exist; there is no essential difference in the specific implementation of the way, so this paper will not be repeated in the experiments on the uphill. Through this experiment, it is verified that SQuRo adopts an efficient closed-loop adaptive motion controller to adapt to the slope environment.

### Collision

**Fig. 9.**
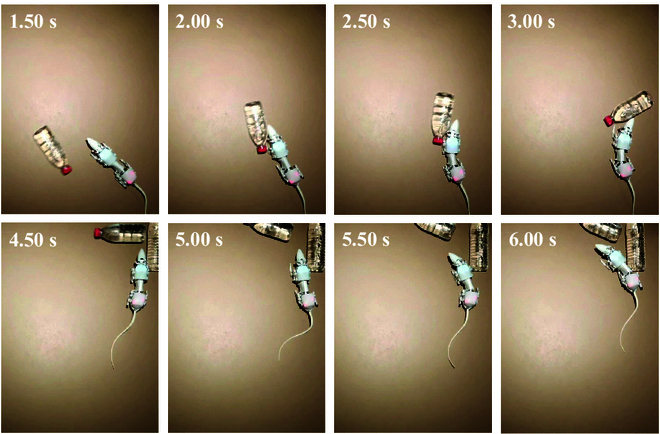
The snapshots of collision experiment.

Obstacle avoidance ability is also very important in evaluating the environmental adaptability of robots. In order to evaluate the ability of SQuRo to detect and avoid obstacles, this study observed the impact detection and avoidance effect of SQuRo through external impact during its operation. As shown in Fig. 9, we rolled 2 water bottles toward the moving robot and made collisions. SQuRo can continue to travel after the first impact at around 1.5 s and adjust its direction according to the impact to avoid obstacles. SQuRo receives a second impact around 4.5 s and adjusts its direction of travel based on the detected impact to avoid obstacles. The experiment verifies SQuRo’s ability of detecting and avoiding external impact by using an efficient closed-loop adaptive motion controller.

## Discussion

The previously proposed bio-inspired design and hierarchical controller enable SQuRo to have superior motion performance in the case of limited computing resources. In this study, we integrate the IMU signal into the control frame and complete the design of the closed-loop adaptive controller. The idea of the controller is to detect whether the robot is in some adverse environment or state through IMU signal and generate adaptive motion for each state. The adaptive motion controller proposed in this paper makes full use of the optimized basic motion modes and does not require the action generated by online calculation, so it is very efficient and can effectively improve the environment adaptability of SQuRo. On the basis of kinematics model and attitude calculation, we achieved the perception of slope angle, attitude, forward direction, accidental fall, and collision. The experimental results show that SQuRo has achieved 6 kinds of robust motion: slope stabilization motion, linear tracking motion, autonomous fall recovery motion, uneven terrain walk, slope walk, and obstacle avoidance, which enhances the robustness of SQuRo in challenging experiments and verifies the effectiveness of closed-loop adaptive motion controller. In order to achieve operation in physically confined spaces, we will continue to improve SQuRo’s motion and robustness. For this purpose, we will further install sensors such as cameras and encoders. In addition, more efficient data fusion and dynamic solvers will be investigated.

This study improved the environmental adaptability of SQuRo and initially established a set of efficient adaptive motion control methods for small-scaled robots. However, at present, state detection and adaptive motion generation can only be carried out for certain situations, and the characteristics and adaptive motion strategies in other environments need to be further studied. Therefore, the next stage of research work will be carried out in the following aspects:

1. Conduct feature analysis on obstacles encountered to achieve autonomous obstacle crossing function.

2. Install microcameras to improve perception in challenging environments and explore control methods for efficient use of visual information.

3. The collaborative movement of the waist and limbs is utilized to further improve the stability of SQuRo during movement.

## Data Availability

All data are incorporated into the article and its online supplementary material.
